# Climate change and primary health care in Chakama, Kilifi County, Kenya

**DOI:** 10.4102/phcfm.v14i1.3670

**Published:** 2022-09-27

**Authors:** Muhsin Sheriff, Robert Mash

**Affiliations:** 1 Centre for Health and Education Programmes, Kilifi, Kenya; 2 Department of Family Medicine and Primary Care, Faculty of Medicine and Health Sciences, Stellenbosch University, Cape Town, South Africa

**Keywords:** climate change, global warming, primary care, primary health care, drought, malnutrition, food insecurity

## Abstract

Chakama is an area of 46 small villages in Kilifi County, Kenya. Climate change has led to more frequent and longer periods of drought in this semi-arid region as well as locust invasions. This has led to a lack of water, with many rivers drying up and poor water quality as a result of pollution of the remaining river water. The lack of water and locust invasion have led to a failure of the crops and loss of livestock. Many pastoralists and farmers have lost their livelihood. Wild animals from local nature reserves have also come into conflict with the community over water scarcity. Many families have migrated in search of water and income. The health effects are seen in the rising number of people suffering from malnutrition and gastroenteritis as well as in terms of mental health problems. Primary health care services are not always available, and the quality of such services is poor. Facilities and healthcare workers also struggle to be resilient in the face of the same environmental challenges. Local nongovernment organisations are attempting to assist through health and social services, community engagement and multisectoral action.

## Context

Chakama is an inland area of around 46 small villages and 4500 households, situated in Kilifi County, Kenya. Kilifi County is situated in the east of Kenya on the Indian Ocean. This is a semi-arid region with low levels of education and literacy. There is just one secondary school, and according to unpublished household data collected by the Centres for Health and Education Programmes (CHEPs), only 6% of people have attended. People undertake subsistence farming and depend on rain to grow maize or support livestock. Poverty is widespread, with difficult access to quality health services.

## Climate change, locust invasion, pollution, health

In sub-Saharan Africa, the frequency of droughts has increased in the last few decades, and they have become more prolonged.^1^ One of the key contributing factors is climate change, leading to reduced rainfall in the region. This in turn has reduced the quantity and quality of water for people. Polluted and contaminated water results in outbreaks of gastroenteritis in populations already weakened by food insecurity. This part of Kenya did not have any rainfall for over 18 months and also suffered an invasion of locusts, which destroyed the crops in early 2020.^2^ The coronavirus disease 2019 (COVID-19) pandemic also restricted movement and led to rising fuel and food prices, which further exacerbated the suffering of the people and limited their ability to access services.

Rivers which were once over one’s head are now trickling at ankle level. Irrigation upstream has further reduced the water volume. The water quality in this river system has deteriorated due to stagnation and contamination with industrial and human pollutants. Rains finally came for about three weeks in December 2021. The next few months again saw no rain, and heat intensity increased. Several older persons in different villages have testified to M.S. that the weather has changed in recent years. ‘I have never experienced such heat and dry spate since my birth’, said Karisa, a 70-year-old man who M.S. met during a community meeting in Hawewanje village. Another man, who walked several hours to the village where M.S. was stationed in to request food, had this to say:

‘Yes, this place is hot and dry, but the intensity this time is definitely different. We have never experienced this kind of drought and heat before. Our suffering this time is much worse.’

Another feature of this story is the impact of the locust invasion at the end of 2019 and beginning of 2020, which devastated crops. This is linked to climate change.^3^ In May 2018, an unusually powerful cyclone hit the Arabian Peninsula, and the heavy rainfall enabled desert locusts to hatch, develop and breed. A further cyclone in October 2018 kept these locusts alive, and they spread to Yemen. From Yemen, the winds of another cyclone in 2019 carried the locusts across the sea to East Africa. Climate change increases the severity and frequency of cyclones through warming of the oceans.^4^

## Human–wildlife conflicts

Compounding these effects are human–wildlife conflicts. Wild animals from the Tsavo National Park have left the park because of the drought and moved into inhabited areas where they destroy crops and come into conflict with humans.^5^ Elephants are often seen where they had not been seen in decades, and they destroy crops grown by hard-working farmers. Hippos congregating in the remaining water are more aggressive, and there have been several attacks and some deaths reported recently. Such conflicts have led to increase in food insecurity, as the farmers cannot work on their land due to the marauding wild animals. The increase in trauma highlights the need for improved emergency transportation and management of trauma at health facilities, which need better training for health workers and medical equipment to deal with such emergencies.

## Migration

Migration of families has also been reported due to the extended drought. One family, comprising of Kesi, her physically challenged husband and five children, recently relocated to another village in the Chakama area to be closer to the river, as their home was in an area that had no water at all. Chakama’s land has become extremely dry, hitting subsistence farmers badly. Grass is depleted, and cows and goats are dying in large numbers, devastating livelihoods, especially those of pastoralists, who totally depend on their livestock. Only those farming near the river are still able to grow some crops, yet many of these too have suffered from elephants moving out of the wildlife parks. The family, with several children, were living on open ground without a house, next to the river. There was no toilet and they were using the bush. The village had no health facility, just like the village they had come from. They wanted to fish and farm the land, to provide food for their family. The mother mentioned that many children in the village had suffered from diarrhoea and vomiting after playing in the river. They had to leave two of their older children behind to attend primary school, as the new area did not have a school. The separation was very stressful, but they wanted their children to complete school and help lift the family out of poverty.

## Food security

Food security has been adversely affected by the changing climate. A lady from Kathama village had this to say, while standing in front of her parched land with evidence of dried crops destroyed by the heat and lack of water.

‘This is my farm. We worked hard on it when the rains finally came after almost two years, but now it’s dry again, and all our efforts have been wasted. We are still going hungry.’

During the frequent visits to the villages in Chakama, M.S. witnessed farm after farm dried up, with large expanses of crops, mainly maize, failed and decimated. Most families here have been pushed to extreme poverty. Many women, especially widows, have been walking long distances searching for support. One widow, who came to the M.S. requesting food and school fees for her children, said:

‘If I had the amount, I would pay for my child. But my crops have been destroyed. That was my only means of livelihood. I am too weak to keep carrying water from the river a few kilometres away. And I have no more cash to rent the pump and buy petrol to pump water from the river to my farm. For two years I have not been able to find casual labour from other farms, as they are all facing the same problems as me.’

## Land use and livelihoods

One source of income that has thrived is making charcoal. Many families have intensified cutting down and burning the bush to make charcoal, which they sell on the roadside to passing cars and lorries. Most of the earnings are used to buy water and maize flour, their staple, and for many this is their only food in days. Cutting down the bush is already showing obvious changes in the environment, which is difficult to restore as there is no culture of growing trees. People are complaining that they have never felt such intense heat in many years. The reduction of bush cover is obvious and there is danger that the consequent loss of the nutrient-rich top soil may negatively impact farming activities and food security. The increasing deforestation leads to the lack of shade with increased risk to livestock of high temperatures.

## Health and health services

There are some public dispensaries, run by nurses, which provide basic medicines and maternity services. The structures have a few consultation rooms, a labour, a delivery room and a pharmacy. When available, trucks fill the water tanks, as water is needed for hygiene, staff accommodation and toilets. There are no surgical or laboratory facilities at this level. Often there are shortages of medicines, and patients are given prescriptions to buy from local chemists. The latter are run by untrained individuals and may provide the wrong medicines from their scarce stock of supplies. The local people often complain of poor-quality care and even mismanagement, and they have to travel to the towns to receive further treatment. Many cannot do so because of the costs involved. These have increased recently because of the fuel crisis and the consequent rise in cost of transport.

There is a network of community health volunteers (CHV) which is activated when there is funding for specific projects, such as polio or measles vaccination campaigns. The CHVs meet at the dispensaries on a monthly basis to discuss various health-related issues. However, many lack insight into public health needs and are poorly motivated to carry out their functions. They are unpaid and have competing demands on personal economic and sociocultural activities, which affect their voluntary work in health services;^6^ hence, their effect on the ground is very limited. There are unmet needs for consistent social and health services to prevent illnesses, promote health and provide quality curative services to the villages. There are gaps in the provision of quality maternal and childcare services. With multiple stressors in the community, there are obvious mental health issues and an increase in alcohol and substance abuse. However, these are not appropriately assessed or documented.

When primary health care services are stretched thin and performing poorly, it is difficult to respond to the health effects of climate change caused by the drought. The resilience of such primary health care facilities and services is low, as they face many of the same climate-related challenges such as the lack of water and high temperatures. There are increasing reports of gastroenteritis from several villages due to the use of contaminated river water, which is at a very low level with flow slowed down, leading to greater concentration of contaminants from upstream villages. The lack of toilets with the common practice of open defaecation worsens the risk of contamination of river water and diseases spread by oro-faecal route. Human–wildlife conflict, especially due to elephants, leading to the destruction of crops in farms which cannot be appropriately fenced due to prevailing extreme poverty, increases food insecurity and risk of malnutrition. Women, children and the elderly are affected most adversely. The government as well as the nongovernment sector have been trying to respond to the crisis.

The CHEPs’ response to the crisis has been multifold.^7^ Firstly, it has expanded its contact with leaders in each of the 46 villages to obtain news of community-wide as well as individual household-level adverse effects. Secondly, it is increasing its capacity to respond through staff training, mentorship and partnerships with similar organisations. The CHEPs keep donors alerted about changing situations and possible needs. Thirdly, village-wide meetings encourage the community to plan and implement their own initiatives to combat the situations they face. Fourthly, resilience-building initiatives have been implemented such as community-led total sanitation, rainwater harvesting, setting up borehole water, desalination plants, village clean water storage, farming training for improved food security and nutrition, various activities to increase school enrolment and quality and adult literacy, as well as community mobilisation for education.

## Conclusion

Chakama in Kilifi County, Kenya, is typical of many such communities in the Horn of Africa that are trying to survive the prolonged drought and effects of climate change. The drought has had many consequences such as the lack of food, the lack of water, poor water quality, conflict with wild animals and migration, which have led to many health effects such as malnutrition, gastroenteritis, trauma and mental health problems. Primary health care services were challenged even before the effects of climate change, and there are significant unmet needs which need to be responded to. Nongovernment organisations, such as CHEPS, have been supporting primary health care through community engagement, multisectoral action and provision of support for health and social services.
